# Psychosocial burden and quality of life in parents of children with anorectal malformation

**DOI:** 10.4103/0971-9261.69135

**Published:** 2010

**Authors:** Gavneet K. Pruthi, Anup Mohta

**Affiliations:** Departments of Clinical Psychology and Pediatric Surgery, Chacha Nehru Bal Chikitsalaya (affiliated to Maulana Azad Medical College), Geeta Colony, Delhi-110031, India

**Keywords:** Anorectal malformation, parents, psychosocial burden, quality of life

## Abstract

**Aim:**

To assess the psychosocial burden and quality of life in parents of children with anorectal malformation.

**Subjects and Methods:**

This is a prospective study conducted in a tertiary care specialty hospital. Sample consisted of 50 parents (care givers), having children with anorectal malformation in the age group of 0-14 years. Data were collected through a semi structured interview with the care givers, using Zarit Burden Interview for assessing extent of burden and WHOQOL-BREF for assessing different aspects of quality of life. Statistical analysis used: frequencies, percentages, mean and standard deviations were calculated for analyzing the data.

**Results:**

The study reveals greater psychosocial burden and poor quality of life, in terms of the psychological and environmental aspects as compared to the physical and social aspects, among the care givers.

**Conclusions:**

The study found increased psychosocial burden and negative impact on the quality of life of parents, which varies with the stages of management and proper continuous counseling is recommended.

## INTRODUCTION

Despite well-executed surgical reconstruction of the anus, many children with high or intermediate ARM may experience functional problems, such as constipation and soiling requiring daily enemas and bowel management programs.[1,2]

The parents play a crucial role in the life of a child suffering from an ARM as their supervision contributes to the degree to which the child learns to cope with his or her disability. Parents of a child suffering from a congenital malformation or chronic illness are liable to suffer from psychosocial disturbances. Parents do have difficulties in coping with the implications of the disorder and express a need for support and that patient care can be improved if aid is tailored to these specific problems.[3]

There has been little work to examine the experiences, negative or positive, of parents of children with chronic conditions, particularly parents of children with anorectal malformations in the Indian setting. The present study is an attempt to assess the psychosocial burden and quality of life in parents of children with anorectal malformation.

## SUBJECTS AND METHODS

The study was carried out at Chacha Nehru Bal Chikitsalaya, Delhi. Sample consisted of 50 parents, without any chronic physical or mental disease, disability or illness, aged 18 years and above having children with confirmed diagnosis of ARM in the age group of 0-14 years. Only those who were staying with their ARM child since birth were enrolled for the study. Furthermore, those who had another child with ARM or any other child with congenital malformation or chronic physical or mental disease, disability or illness were excluded from the study.

Data were collected by conducting the semi-structured interview. The parents were interviewed by a qualified Clinical Psychologist (the first author). Before the collection of the data, informed consent was taken from the participants (parents); and they were assured about the confidentiality. Wherever necessary, the tools were translated into Hindi, and with the help of examples, they were simplified and administered to the illiterate parents (caregivers). Information gathering started with preformed Basic Data Identification Schedule and then the following tools were administered:

- Zarit Burden Interview:[4] The interview schedule contains 22 items, and for each of the 22 items, caregivers were asked to respond about the impact of the patient’s illness on their life, by indicating how often they felt in a particular way (“never,” “rarely,” “sometimes,” “quite frequently,” or “nearly always”).
- WHOQOL-BREF:[5] The tool consists of 26 items and is used to measure quality of life. The items are rated on a 5-point scale, which gives a profile with 4 domain scores (physical health, psychological health, social relationship, and environment) and 2 individually scored items about an individual’s overall perception of quality of life and health (Q1 and Q2). The 4 domains are scaled in positive direction, with a score range of 0-100; and higher score denoting higher quality of life.

## RESULTS

### Description of the identified sample

The study included parents of 50 children-31 males and 19 females, suffering with ARM. The educational status of the parents included 7 (illiterate), 32 (undergraduate), 6 (graduate), and 5 (postgraduate). The age of the children ranged from 4 days to 11 years and most of the children were in the age group of 2.5-5 years. The age of the parents ranged from 20-35 years with maximum parents in the age group 22-25 years. Monthly income of the parents ranged from Rupees 200-50,000 with maximum parents in the monthly income group Rs 3000-5000 per month. Most parents belonged to lower socioeconomic strata.

### Psychosocial burden

In the sphere of psychosocial burden, maximum parents perceived mild level of burden [Table 1]. Also through, semi-structured interview, it was found that many children had undergone 2-3 surgical procedures by the time of interview; therefore, they could adjust to the procedures and its effects.

**Table 1:** Frequency of parents in each of the category of burden for the Zarit Burden Interview

Category	Little	Mild	Moderate	Severe
Frequency (%), N = 50	11 (22)	24 (48)	10 (20)	5 (10)

### Quality of life

With reference to quality of life, the results of the study reveal that the overall perception of quality of life (WHOQOL-BREF, Item no. 1) was “neither poor, neither good” [Table 2] and overall perception of health (WHOQOL-BREF, Item 2), was reported as “neither satisfied nor dissatisfied” [Table 3], for the present sample in most parents.

**Table 2:** Frequency of parents in each of the category for WHOQOL-BREF, Item no. 1 (overall quality of life)

Category	Very poor	Poor	Neither poor nor good	Good	Very good
Frequency (%), N = 50	5 (10)	11 (22)	29 (58)	3 (6)	2 (4)

**Table 3:** Shows the frequencies and percentage for the categories of WHOQOL-BREF; Item no. 2 (overall perception of health)

Category	Very dissatisfied	Dissatisfied	Neither satisfied nor dissatisfied	Satisfied	Very satisfied
Frequency (%), N = 50	3 (6)	5 (10)	22 (44)	16 (32)	4 (8)

The mean and standard deviation (SD) scores for quality of life and overall perception of health were 2.95 (0.37) and 3.06 (0.9), respectively. Although quality of life and overall perception of health were found to be neither good nor poor, overall perception of quality of life was found to be poorer than overall perception of health, among the present sample of caregivers. During the semi-structured interview also, it was reported by the caregivers that they get more stressed and strained, by thinking about the child’s future rather than by taking care of themselves.

### Four domains of quality of life

Among the 4 domains of WHOQOL-BREF, the results of the study revealed, quality of life to be poorest in the psychological domain and best in the social domain [Table 4]. The mean scores for the psychological domain was found to be lowest, but much difference was not found among the mean scores for psychological, environmental, and physical domains, suggesting that they were equally affected, whereas the social domain was not much affected. During the semi-structured interview also, it was found that, caregivers were more stressed and strained about their child’s present condition and about their financial condition and means of transport.

**Table 4:** Mean (standard deviation) scores for different domains of WHOQOL-BREF

Domain	Physical	Psychological	Social	Environmental
Mean score and standard deviation	*x* = 41.85 σ = 8.96	*x* = 34.6 σ = 9.37	*x* = 48.75 σ = 14.49	*x* = 38.85 σ = 7.35

## DISCUSSION

Anorectal malformations are common congenital anomalies that have a wide spectrum of defects. Low anorectal anomalies can be managed by simple perineal procedures but high and intermediate anomalies need staged multiple surgical procedures for correction. After birth, a preliminary colostomy is frequently required followed by definitive procedure at a later age. Anal dilatation is necessary to prevent stenosis of the neoanus. The child may require interventions, such as regular enemas, washouts, or procedures, such as antegrade colonic enemas for management of constipation or fecal incontinence.[1,2]

Birth and hospitalization of a baby with a congenital anomaly requiring surgery at birth is a demanding and emotionally stressing experience for a couple. It has been noticed that having a child with a chronic illness or congenital malformation leads to heavy impact on family members. The consequences of chronic childhood diseases cause disturbances in the mental health of parents. Parents have to deal not only with their child’s disease but also need to follow the prolonged therapy schedule, which could be as distressing as the disease itself.[6] The social and financial aspects of life may be difficult for parents of a child with a chronic condition.[7] Managing stomas, postoperative dilatation, and enemas can lead to physical, psychosocial, emotional, and social problems in the parents causing alterations in their psychosocial milieu.[8,9] Even among the parents, mothers may suffer more than the fathers as it has been shown that responsibility of managing the child’s bowel habits and incontinence is often taken by the mother who most likely performs the regular enemas causing high level of anxiety associated with the child’s ARM and the extensive follow-up treatments.[10,11]

The present study has addressed the issue of psychosocial burden and quality of life in parents having children with ARM. Results indicated that most parents suffered only mild level of psychosocial burden, which has been demonstrated in earlier studies also. Nissell *et al*.[12] demonstrated the positive experiences reported by the parents with reference to the child development, parental development, and family strengthening. Using Nijmegen Questionnaire on Child-rearing Situations, Child Behavior Checklist, and the Teacher Report Form, Hassink *et al*.[3] investigated the stress in parenting a child with ARM and also attempted to identify somatic or behavioral characteristics in the child that influence the stress of parenting. They concluded that the perception of parenting stress is not different in parents of children with an ARM as compared with those with healthy primary school children. Findings in consonance with the same were found in the present study, that is, most of the parents reported only mild level of psychosocial burden.

Quality of life of the parents was also affected in the present study. The expressed areas of concern were feelings of despair, anxiety, depression, meaninglessness of life, present health services, amount of information provided to them, the means of transport, and their financial conditions rather than the social aspects. The last two concerns are important considering the low socioeconomic status of most parents in the present study. More intensive family support may be indicated for those with minority group or low socioeconomic status, limited social support, or high perceived burden.[13] Psychosocial variables also contribute to the development of psychopathology and thereby determine outcomes for global well-being and health-related quality of life.[14]

Parents need to have better information about the congenital malformation, its management, and long-term consequences on the day-to-day life.[15] This is especially so when the child with ARM suffers from postoperative constipation or fecal incontinence. Failure of the clinicians to understand the needs and provide necessary support may lead to dissatisfaction among the parents.[16] Sometimes the difficult behavior of the child also adds to the stress of the parents. Poor quality of life in children as interpreted by the parents also has an impact on the parents’ psychosocial well-being.[17]

The surgeon should well address the parents’ informational needs in the immediate perioperative period.[15] Parents need information on not only the child’s malformation and the surgery but also the functional prognosis in the later life. It is at this stage that the parents feel the need for appropriate psychological support.[18] Support to parents in caring for a child with ARM should be individualized and occasionally undertaken through collaboration with experts from child and adolescent psychiatry.[12] Innovative psychosocial intervention programs are needed in primary care settings to reduce family needs and promote child health. Clinicians should keep in mind that having a child with ARM does not automatically imply that the parents experience child-rearing problems,[3] but constant support is necessary to prevent increased psychosocial burden on the parents.

## CONCLUSION

The present study concludes that there is greater psychosocial burden and poor quality of life among parents of children with ARM. It is imperative to provide psychosocial support, including promotion of a clear understanding of the disease to these participants. However, our results should be interpreted in the light of small sample size and more studies with large sample sizes and statistical tests employing indepth analysis are required.
